# Draft genome sequences of three fungal-interactive *Paraburkholderia terrae* strains, BS007, BS110 and BS437

**DOI:** 10.1186/s40793-017-0293-8

**Published:** 2017-12-18

**Authors:** Akbar Adjie Pratama, Irshad Ul Haq, Rashid Nazir, Maryam Chaib De Mares, Jan Dirk van Elsas

**Affiliations:** 1 Department of Microbial Ecology, Microbial Ecology - Groningen Institute for Evolutionary Life Sciences, University of Groningen, Nijenborgh 7, Groningen, 9747 AG The Netherlands; 2 Department of Environmental Sciences COMSATS Institute of Information Technology, University Road, Abbottabad, 22060 Pakistan

**Keywords:** *Paraburkholderia terrae*, Mycosphere, Fungal-interactive, Genome

## Abstract

Here, we report the draft genome sequences of three fungal-interactive 10.1601/nm.27008 strains, denoted BS110, BS007 and BS437. Phylogenetic analyses showed that the three strains belong to clade II of the genus 10.1601/nm.1619, which was recently renamed 10.1601/nm.26956. This novel genus primarily contains environmental species, encompassing non-pathogenic plant- as well as fungal-interactive species. The genome of strain BS007 consists of 11,025,273 bp, whereas those of strains BS110 and BS437 have 11,178,081 and 11,303,071 bp, respectively. Analyses of the three annotated genomes revealed the presence of (1) a large suite of substrate capture systems, and (2) a suite of genetic systems required for adaptation to microenvironments in soil and the mycosphere. Thus, genes encoding traits that potentially  confer fungal interactivity were found, such as type 4 pili, type 1, 2, 3, 4 and 6 secretion systems, and biofilm formation (PGA, alginate and *pel*) and glycerol uptake systems. Furthermore, the three genomes also revealed the presence of a highly conserved five-gene cluster that had previously been shown to be upregulated upon contact with fungal hyphae. Moreover, a considerable number of prophage-like and CRISPR spacer sequences was found, next to genetic systems responsible for secondary metabolite production. Overall, the three 10.1601/nm.27008 strains possess the genetic repertoire necessary for adaptation to diverse soil niches, including those influenced by soil fungi.

**Electronic supplementary material:**

The online version of this article (10.1186/s40793-017-0293-8) contains supplementary material, which is available to authorized users.

## Introduction

The genus 10.1601/nm.1619 was proposed in 1993 by Yabuuchi et al. [1]. Following this, continuing emendation of the genus has occurred, mainly as a result of the addition of new species. Recent molecular and phylogenetic analysis of the genus divided it into two clades, with clade I containing the pathogenic 10.1601/nm.1619 spp. and clade II mainly environmental bacteria. The latter clade was reclassified as a novel genus, named 10.1601/nm.26956 [2, 3]. This genus encompasses a suite of highly diverse and environmentally adaptable bacteria that are able to occupy various ecological niches, ranging from soil [4, 5] to plants and humans [6]. Members of the genus 10.1601/nm.26956 are also known to harbor some of the largest genomes among all known bacteria [7, 8].

10.1601/nm.27008 strain BS001, which was isolated as a co-migrator in soil with the saprotrophic fungus *Lyophyllum sp.* strain Karsten [9], has been extensively described, and it is used here as a reference organism. *P. terrae* strain BS110 was isolated from the mycosphere of the ecotomycorrhizal fungus *Laccaria proxima* [5, 9] and also showed comigration capacity with the aforementioned fungus. The other two 10.1601/nm.27008 strains (BS007, BS437) were isolated – similarly – as mycosphere dweller / comigrator, from soils collected in Gieterveen and Wageningen, the Netherlands, respectively [5, 9]. Being avid mycosphere inhabitants, all these 10.1601/nm.26956 strains might play essential roles in the ecology of soil fungi and so in (degradative) ecosystem functions. Several studies have been performed to address such interactions and understand the mechanisms involved. An in-depth study of the genome of 10.1601/nm.27008 strain BS001 revealed its remarkable genetic potential, including genetic systems that presumably enable it to interact with saprotrophic fungi like *Lyophyllum* sp. strain Karsten [5, 8]. Moreover, the strain BS001 genome was found to contain numerous regions of genomic plasticity that are typified by different plasmid- and prophage-like genes [8]. We took this finding as a token of the remarkable ability of 10.1601/nm.27008 to adapt – via horizontal gene transfer - to fluctuating local challenges, including the presence of fungal counterparts. The strategies that are presumably used in this fungal interactivity include (but are not limited to): (i) biofilm formation on fungal surfaces [9, 10], (ii) a type-3 secretion system (T3SS) with a subtle role in the cellular migration along fungal hyphae and adherence [10, 11] and (iii) chemotaxis towards growing fungal hyphae and subsequent adherence to fungal surfaces [10]. In a recent study, it was shown that *P. terrae* strain BS001 differentially expresses genes involved in chemotaxis, flagellar motility and metabolic and stress response mechanisms in response to fungal hyphae [12].

Given the fact that the three novel 10.1601/nm.27008 strains BS110, BS437 and BS007 were isolated by virtue of their capacity to interact with soil fungi, we hypothesized that their physiological responses to fungi, as reflected in their genomic make-up, might be similar across them and akin to those of the well-studied strain BS001. To further explore this tenet, analyses of sequenced genomes constitute a necessary first step. Here, we present a summary of the draft genome sequences, and their annotation, of the three novel 10.1601/nm.27008 strains. Furthermore, we examine the traits that allow to build hypotheses with respect to the ecological relevance of these strains in the mycosphere, coupled to analyses of phenotypes. Based on these characteristics, we thus shed light on the potential strategies that these strains may use in the interplay with their fungal counterparts.

## Organism information

### Classification and features

**Fig. 1:**
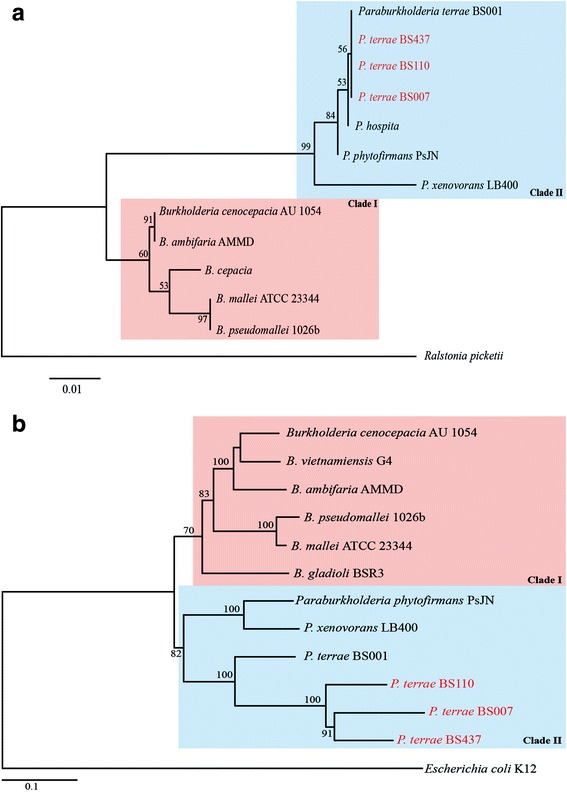
Phylogenetic tree of selected 10.1601/nm.1619and *Paraburkholderia* strains based on 16S rRNA gene sequences (**a**) and on alignment of seven concatenated core genes (*aroE, dnaE, groeL, gyrB, mutL, recA,* and *rpoB*) (**b**). Evolutionary distance were computed with MEGA7 using the maximum-likelihood method. The bootstrap values above 50% (from 1000 replicates) are indicated at the nodes. 10.1601/nm.27008 strains BS007, BS110 and BS437 were all found to  belong to clade II. Clade I mainly consists of pathogenic 10.1601/nm.1619species, while clade II, mainly consisting of environmental strains, was assigned to the new genus 10.1601/nm.26956. See Sawana *et al*. [3]

10.1601/nm.27008 BS110 and BS007 were isolated from the base of fruiting bodies of the ectomycorrhizal fungus *Laccaria proxima*, sampled in Gieterveen, the Netherlands [9]. Like the reference strain BS001, strain BS437 was isolated as a comigrator with *L.* sp strain Karsten (in this case it was isolated from  soil from Droevendaal, Wageningen, the Netherlands). The collected samples were treated as previously described [5, 9]. Briefly, for isolation of 10.1601/nm.27008 BS110 and BS007, mycosphere samples were carefully collected from soil adhering to the dense *L. proxima* hyphae just below the fruiting body. Strains BS001 and BS437 were isolated as ‘winners’ of microbiome co-migration experiments [5, 9]. All isolated 10.1601/nm.26956 strains were grown on LB medium at 28 °C. Phylogenetic analyses based on alignment of seven concatenated core genome genes (*aroE, dnaE, groeL, gyrB, mutL, recA,* and *rpoB*) (Fig. 1) showed that 10.1601/nm.27008 strains BS110, BS007 and BS437 clustered within the 10.1601/nm.26956 genus (akin to the former 10.1601/nm.1619 clade II), as reported previously for strain BS001 [8]. Based on these analyses, our four *P. terrae* strains were also found to be closely related to 10.1601/nm.26993 and 10.1601/nm.27015 *.*

**Fig. 2:**
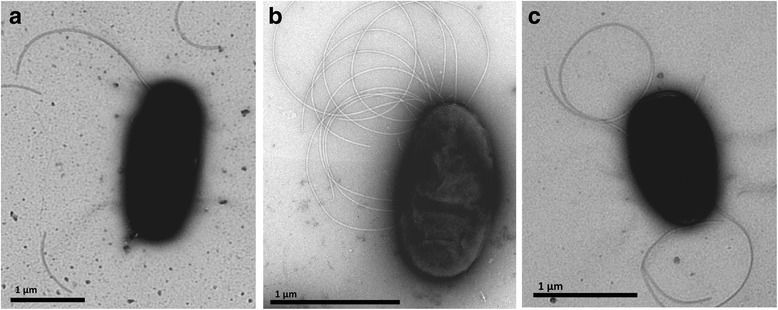
Transmission electron microscopy of (**a**) 10.1601/nm.27008 strain BS110, (**b**) *P. terrae  *strain BS007, and (**c**) *P. terrae  *strain BS437. The scale bars represent 1 μm

Gram staining of freshly-grown cells of10.1601/nm.27008 strains BS007, BS110 and BS437 revealed all three strains to be  Gram-negative. Transmission electron microscopy of freshly-grown cultures showed that each strain population consisted mainly of single cells that were rod-shaped (cell lengths 1 to 2 μm), with predominantly polar flagella (Fig. 2).

The growth of all strains was tested at different temperatures (4, 12, 15, 18, 24, 37, 42 and 50 °C). For all strains, the temperature range that allowed the formation of detectable CFUs on plates was 15-37 °C, with optimum growth being recorded at 28 °C within 3 days. The pH tolerance of strains was tested by assessing the growth of colonies of each of the strains on R2A plates under different pH (specifically 4.0, 5.0, 6.0, 7.0, 8.0, 9.0 and 10.0) at 28 °C. All strains were able to grow in the pH range 5.0–10.0, with optimum growth at pH 6.0–7.0. No growth was recorded at pH 4.0. Salt tolerance assays were done by placing cells on R2A plates supplemented with different NaCl concentrations (specifically zero, 0.5, 1.0, 2.0, 2.5, 5.0 and 10%), and incubating for up to five days, with regular observation of colony formation. Strains BS007, BS110 and BS437 were able to grow at up to 1% NaCl in the R2A medium, being strongly inhibited at 2% NaCl. Hence, all three strains tested are quite salt-sensitive.

The capacities of the strains to utilize an array of carbon sources were tested using BIOLOG GN2 assays (Biolog Inc., Hayward, CA). The results revealed that most strains are able to utilize a suite of different carbonaceous compounds (Tables 1, 2, and 3) (as in Nazir *et al*. [5]). Some of the carbonaceous compounds could only be utilized by some, but not all, strains. That is, strains BS007 and BS110 (but not BS437) could utilize d-trehalose, phenyl ethyl-amine, 2,3-butanediol and gentiobiose. The compound d-cellobiose was utilized only by strains BS007 and BS437, while γ-hydroxybutyric acid was utilized only by strains BS110 and BS437. There was also substrate specificity, in that some compounds could only be utilized by one strain each. For instance, strain BS007 utilized itaconic acid, whereas d-serine and α-d-lactose were uniquely utilized by strain BS110, and d-melibiose, β-methyl-d-glucoside and α-ketoglutaric acid by strain BS437.

**Table 1:** Classification and general features of 10.1601/nm.27008 strain BS110 [26]

MIGS ID	Property	Term	Evidence code^a^
		Domain: Bacteria	TAS [27]
		Phylum: 10.1601/nm.808	TAS [28]
		Class: 10.1601/nm.1616	TAS [29]
		Order: 10.1601/nm.1617	TAS [30]
		Family: 10.1601/nm.1618	TAS [31]
		Genus: 10.1601/nm.26956	TAS [3, 32]
		Species: 10.1601/nm.27008	TAS [3, 32]
		Strain: BS110	TAS [5]
	Gram-stain	Negative	IDA, TAS [5, 32]
	Cell shape	Rod-shaped	IDA, TAS [5, 32]
	Motility	Motile	TAS [5, 32]
	Sporulation	Not reported	
	Temperature range	15 °C − 37 °C	TAS [5]
	Optimum temperature	28 °C	TAS [5]
	pH range; Optimum	5.0–10.0; 6.0–7.0	TAS [5]
	Carbon source	Tween40, tween80, l-fucose, gentiobiose, α-d-lactose, lactulose, d-psicose, d-trehalose, xylitol, succinic acid monomethyl ester, γ- hydroxybutyric acid, itaconic acid, α-ketovaleric acid, succinamic acid, glucuronamide, l-alaninamide, d-alanine, l-ornithine, d-serine, d,l-carnitine, urocanic acid, phenylethyl-amine, 2,3-butanediol, d,l, α- glycerol phosphate, d-glucose-6-phosphate	TAS [5]
MIGS-6	Habitat	Soil, mycosphere	TAS [5, 32]
MIGS-6.3	Salinity	1% NaCl	TAS [5]
MIGS-22	Oxygen requirement	Aerobic	TAS [5]
MIGS-15	Biotic relationship	Soil microbe, free living	TAS [5]
MIGS-14	Pathogenicity	Non pathogen	TAS [5]
	Biosafety level	Non pathogen	TAS [5]
MIGS-15	Geographic location	Gieterveen, Netherlands	TAS [5]
MIGS-5	Sample collection	2012	TAS [5]
MIGS-4.1	Latitude	53° N	TAS [5]
MIGS-4.2	Longitude	6° E	TAS [5]

^a^Evidence codes - *IDA* Inferred from Direct Assay, *TAS* Traceable Author Statement (i.e., a direct report exists in the literature), *NAS* Non-traceable Author Statement (i.e., not directly observed for the living, isolated sample, but based on a generally accepted property for the species, or anecdotal evidence). These evidence codes are from the Gene Ontology project. If the evidence is IDA, then the property was directly observed for a live isolate by one of the authors or an expert mentioned in the acknowledgement

**Table 2:** Classification and general features of 10.1601/nm.27008 strain BS007 [26]

MIGS ID	Property	Term	Evidence code^a^
		Domain: Bacteria	TAS [27]
		Phylum: 10.1601/nm.808	TAS [28]
		Class: 10.1601/nm.1616	TAS [29]
		Order: 10.1601/nm.1617	TAS [30]
		Family: 10.1601/nm.1618	TAS [31]
		Genus: 10.1601/nm.26956	TAS [3, 32]
		Species: 10.1601/nm.27008	TAS [3, 32]
		Strain: BS007	TAS [5]
	Gram-stain	Negative	IDA, TAS [5, 32]
	Cell shape	Rod-shaped	IDA, TAS [5, 32]
	Motility	Motile	TAS [5, 32]
	Sporulation	Not reported	
	Temperature range	15 °C − 37 °C	TAS [5]
MIGS	Optimum temperature	28 °C	TAS [5]
	pH range; Optimum	5.0–10.0; 6.0–7.0	TAS [5]
	Carbon source	Tween40, tween80, d-cellobiose, l-fucose, gentiobiose, lactulose, d-psicose, d-trehalose, xylitol, succinic acid monomethyl ester, itaconic acid, α-ketovaleric acid, succinamic acid, glucuronamide, l-alaninamide, d-alanine, l-ornithine, d,l-carnitine, urocanic acid, phenylethyl-amine, 2,3-butanediol, d,l, α- glycerol phosphate, d-glucose-6-phosphate	TAS [5]
MIGS-6	Habitat	Soil, mycosphere	TAS [5, 32]
MIGS-6.3	Salinity	1% NaCl	TAS [5]
MIGS-22	Oxygen requirement	Aerobic	TAS [5]
MIGS-15	Biotic relationship	Soil microbe, free living	TAS [5]
MIGS-14	Pathogenicity	Non pathogen	TAS [5]
	Biosafety level	Non pathogen	TAS [5]
MIGS-15	Geographic location	Gieterveen, Netherlands	TAS [5]
MIGS-5	Sample collection	2012	TAS [5]
MIGS-4.1	Latitude	53° N	TAS [5]
MIGS-4.2	Longitude	6° E	TAS [5]

^a^Evidence codes - *IDA* Inferred from Direct Assay, *TAS* Traceable Author Statement (i.e., a direct report exists in the literature), *NAS* Non-traceable Author Statement (i.e., not directly observed for the living, isolated sample, but based on a generally accepted property for the species, or anecdotal evidence). These evidence codes are from the Gene Ontology project. If the evidence is IDA, then the property was directly observed for a live isolate by one of the authors or an expert mentioned in the acknowledgement

**Table 3:** Classification and general features of 10.1601/nm.27008 strain BS437 [26]

MIGS ID	Property	Term	Evidence code^a^
		Domain: Bacteria	TAS [27]
		Phylum: 10.1601/nm.808	TAS [28]
		Class: 10.1601/nm.1616	TAS [29]
		Order: 10.1601/nm.1617	TAS [30]
		Family: 10.1601/nm.1618	TAS [31]
		Genus: 10.1601/nm.26956	TAS [3, 32]
		Species: 10.1601/nm.27008	TAS [3, 32]
		Strain: BS437	TAS [5]
	Gram-stain	Negative	IDA, TAS [5, 32]
	Cell shape	Rod-shaped	IDA, TAS [5, 32]
	Motility	Motile	TAS [5, 32]
	Sporulation	Not reported	
	Temperature range	15 °C − 37 °C	TAS [5]
	Optimum temperature	28 °C	TAS [5]
	pH range; Optimum	5.0–10.0; 6.0–7.0	TAS [5]
	Carbon source	Tween40, tween80, d-cellobiose, l-fucose, α-d-lactose, lactulose, d-melibiose, β-methyl-d-glucoside, d-psicose, xylitol, succinic acid monomethyl ester, γ- hydroxybutyric acid, α-ketoglutaric acid, α-ketovaleric acid, succinamic acid, glucuronamide, l-alaninamide, d-alanine, l-ornithine, d,l-carnitine, urocanic acid, 2,3-butanediol, d,l, α- glycerol phosphate, d-glucose-6-phosphate	TAS [5]
MIGS-6	Habitat	Soil, mycosphere	TAS [5, 32]
MIGS-6.3	Salinity	1% NaCl	TAS [5]
MIGS-22	Oxygen requirement	Aerobic	TAS [5]
MIGS-15	Biotic relationship	Soil microbe, free living	TAS [5]
MIGS-14	Pathogenicity	Non pathogen	TAS [5]
	Biosafety level	Non pathogen	TAS [5]
MIGS-15	Geographic location	Wageningen, Droevendaal, Netherlands	TAS [5]
MIGS-5	Sample collection	2012	TAS [5]
MIGS-4.1	Latitude	52° N	TAS [5]
MIGS-4.2	Longitude	5° E	TAS [5]

^a^Evidence codes - *IDA* Inferred from Direct Assay, *TAS* Traceable Author Statement (i.e., a direct report exists in the literature), *NAS* Non-traceable Author Statement (i.e., not directly observed for the living, isolated sample, but based on a generally accepted property for the species, or anecdotal evidence). These evidence codes are from the Gene Ontology project. If the evidence is IDA, then the property was directly observed for a live isolate by one of the authors or an expert mentioned in the acknowledgement

## Genome sequencing information

### Genome project history

10.1601/nm.27008 BS110 and BS007 were isolated from the base of fruiting bodies of *Laccaria proxima*, in Gieterveen, the Netherlands and strain BS437 was isolated - as a co-migrator with *L.* sp strain Karsten - from Droevendaal, Wageningen, The Netherlands. The three strains were selected for sequencing, as they showed migration proficiency in soil along with the fungus *Lyophyllum* sp. strain Karsten, similar to the closely related 10.1601/nm.27008 strain BS001 [5]. Moreover, there is a current lack of knowledge on the mechanisms behind the behavior of such fungal-interactive 10.1601/nm.27008 strains. Sequencing of the draft genomes was completed in 2012, and the sequences of strain BS007, BS110 and BS437 have been deposited for public release at NCBI under the accession numbers NFVE00000000, NFVD00000000 and NFVC00000000, respectively. A summary of the project information is shown in Table 4.

**Table 4:** Project information

MIGS ID	Property	Strain BS110 term	Strain BS007 term	Strain BS437 term
MIGS 31	Finishing quality	Draft genome	Draft genome	Draft genome
MIGS-28	Libraries used	Illumina TruSeq libraries	Illumina TruSeq libraries	Illumina TruSeq libraries
MIGS 29	Sequencing platforms	Illumina HiSeq2000	Illumina HiSeq2000	Illumina HiSeq2000
MIGS 31.2	Fold coverage	200.37	224.16	241.39
MIGS 30	Assemblers	Velvet version 1.2.05	Velvet version 1.2.05	Velvet version 1.2.05
MIGS 32	Gene calling method	MicroScope Genoscope platform [13]	MicroScope Genoscope platform [13]	MicroScope Genoscope platform [13]
	Locus Tag	BTR	BTI	BTS
	Genbank ID	NFVD00000000	NFVE00000000	NFVC00000000
	GenBank Date of Release	24 May 2017	24 May 2017	24 May 2017
	GOLD ID	Gp0216754	Gp0216770	Gp0216771
	BIOPROJECT	PRJNA385388	PRJNA385388	PRJNA385388
MIGS 13	Source Material Identifier	SAMN06888377 *Paraburkholderia* collection of The Department of Microbial Ecology, University of Groningen, Netherlands (RUGME_B3G4)	SAMN06888376 *Paraburkholderia* collection of The Department of Microbial Ecology, University of Groningen, Netherlands (RUGME_B3F6)	SAMN06888378 *Paraburkholderia* collection of The Department of Microbial Ecology, University of Groningen, Netherlands (RUGME_B3H4)
	Project relevance	Fungi- interactive, phylogenetic tree, prophage identification.	Fungi- interactive, phylogenetic tree, prophage identification.	Fungi- interactive, phylogenetic tree, prophage identification.

### Growth conditions and genomic DNA preparation

All strains were grown aerobically on LB medium at 28 °C (180 rpm, shaking, overnight). The genomic DNA of the overnight cultures was then extracted using a modified (Powersoil) DNA isolation kit (MOBio Laboratories Inc., Carlsbad, CA, USA). The modification consisted of adding glass beads to the cultures to spur mechanical cell lysis. This extraction method is a rapid way to produce highly pure DNA from bacterial cultures. The extracted gDNAs were purified with the Wizard DNA cleanup system (Promega, Madison, USA). The quality and quantity of the extracted DNAs were assessed using electrophoresis in 1% agarose.

### Genome sequencing and assembly

The genomic DNAs of 10.1601/nm.27008 strains BS110, BS007 and BS437 were sequenced on the Illumina HiSeq2000 platform by LCG Genomics (Berlin, Germany). The libraries for the strains were prepared using Illumina TruSeq libraries with Covaris-sheared DNA or TruSeq® Nano DNA Library Prep. Totals of approximately 18, 16 and 17 million paired reads were produced for the 10.1601/nm.27008 BS007, BS110 and BS437 strains, respectively. Illumina’s CASAVA data analysis software was used for further processing, such as adapter trimming and quality trimming using the fastX toolkit. K-mer error correction analysis was done using Quake Version 0.3; the K-mer corrected paired reads were 16, 15 and 15 million for BS007, BS110 and BS437. Genome assembly was then carried out using Velvet version 1.2.05, by LCG Genomics (statistics of the sequencing is provided in Additional file 1: Table S1). Totals of 788, 658 and 843 contigs were formed following assembly, for strains BS007, BS110 and BS437, respectively.

The 16S rRNA genes were extracted and added as a separate scaffold. The extraction of 16S rRNA genes was done using SortMeRNA and assembly using SPAdes version 3.9.0.

### Genome annotation

The sequence information of the 10.1601/nm.27008 BS007, BS110 and BS437 genomes was submitted to the MicroScope platform that is hosted at Genoscope [13] for analysis. The gene annotation editor in MicroScope was used; it includes the use of TrEMBL, SwissProt alignments, the PubMed and InterPro databases and SignalP. The MicroScope platform is also integrated with a metabolic profiling platform that includes the PkGDB database, as well as MicroCyc that is designed to extract genomic and metabolic data from the Pathway Genome Databases, KEGG and the secondary metabolite detection program antiSMASH [13].

## Genomic properties

**Fig. 3:**
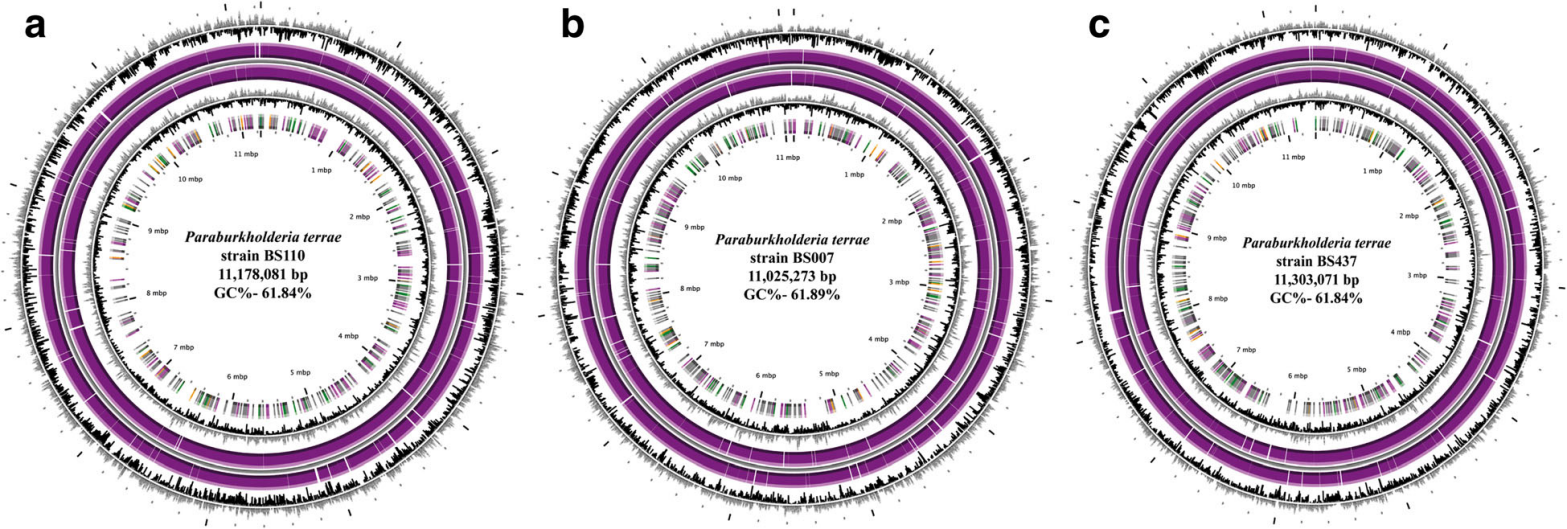
Circular view of genome sequences (each consisting of several replicons) of *Paraburkholderia terrae* (**a**) strain BS110, (**b**) strain BS007 and (**c**) strain BS437. The circular display shows, from outside to inside: (i) GC percentage; (ii) Predicted CDSs transcribed in the clockwise direction; (iii) Predicted CDSs transcribed in the counterclockwise direction. (purple colour in (2) and (3) represents Primary/Automatic annotations), (iv) GC skew (G + C/G-C) and (v) color-code representing rRNA (blue), tRNA (green), miscellaneous RNA (orange), Transposable elements (pink) and pseudogenes (grey)

The genome of strain BS007 has an estimated size of 11,025,273 bp, with 61.89% G + C content, that of strain BS110 11,178,081 bp (61.84% G + C), and that of strain BS437 11,303,071 bp (61.84% G + C) (Fig. 3). The three genomes contain 10,411 (86.83%), 10,288 (85.85%) and 10,610 (86.03%) protein-encoding regions, respectively. The properties and statistics of the genomes are summarized in Table 5, and the numbers of genes associated with general COG functional categories in Table 6.

**Table 5:** Genome statistics

Attribute	Strain BS110		Strain BS007		Strain BS437	
	Value	% of total^a^	Value	% of total^a^	Value	% of total^a^
Genome size (bp)	11,178,081	100	11,025,273	100	11,303,071	100
Coding DNA (bp)	9,596,382	85.85	9,573,245	86.83	9,724,031	86.03
DNA G + C content	6,912,525	61.84	6,823,541	61.89	6,983,037	61.78
DNA Scaffolds	658	–	788	–	843	–
Total genes	11,984	100	11,991	100	12,333	100
Protein encoding genes	10,288	85.85	10,411	86.83	10,610	86.03
RNA genes	54		48		53	
Pseudogenes	N/D	–	N/D	–	N/D	–
Genes in internal clusters	N/D	–	N/D	–	N/D	–
Genes with function prediction	4458	37.2	4461	37.2	4743	38.46
Genes assigned to COGs	8327	69.49	8273	69	8465	68.64
Genes assigned Pfam domains	4015	33,50	3857	32.17	4106	33.29
Genes with signal peptides	976	8.14	1001	8.35	1053	8.53
Genes with transmembrane helices	1592	13,28	1555	12.97	1632	13.23
CRISPR spacers	22		21		15	

^a^The total is based on either the size of the genome in base pairs or the total number of protein encoding genes in the annotated genome; *N/D* not determined

**Table 6:** Number of genes associated with general COG functional categories of 10.1601/nm.27008 strain BS110, BS007, and BS437

Code	Strain BS110		Strain BS007		Strain BS437		Description
	Value	% of total^a^	Value	% of total^a^	Value	% of total^a^	
J	242	2.03	242	2.03	253	2.06	Translation, ribosomal structure and biogenesis
A	1	0.008	1	0.008	1	0.008	RNA processing and modification
K	1030	8.65	1030	8.65	1026	8.37	Transcription
L	422	3.54	422	3.54	443	3.61	Replication, recombination and repair
B	4	0.03	4	0.03	4	0.03	Chromatin structure and dynamics
D	68	0.57	68	0.57	69	0.56	Cell cycle control, cell division, chromosome partitioning
V	95	0.79	95	0.79	104	0.84	Defense mechanisms
T	573	4.81	573	4.8	605	4.93	Signal transduction mechanisms
M	547	4.59	547	4.59	553	4.51	Cell wall/membrane biogenesis
N	161	1.35	161	1.35	172	1.4	Cell motility
U	202	1.69	202	1.69	210	1.71	Intracellular trafficking and secretion
O	267	2.24	267	2.24	271	2.21	Posttranslational modification, protein turnover, chaperones
C	774	6.5	774	6.5	793	6.47	Energy production and conversion
G	784	6.58	784	6.58	769	6.27	Carbohydrate transport and metabolism
E	1193	10.02	1193	10.02	1188	9.69	Amino acid transport and metabolism
F	114	0.96	114	0.96	109	0.89	Nucleotide transport and metabolism
H	263	2.21	263	2.21	268	2.18	Coenzyme transport and metabolism
I	461	3.87	461	3.87	476	3.88	Lipid transport and metabolism
P	706	5.93	706	5.93	713	5.81	Inorganic ion transport and metabolism
Q	387	3.25	387	3.25	395	3.22	Secondary metabolite biosynthesis, transport and catabolism
R	1496	12.57	1496	12.57	1544	12.59	General function prediction only
S	682	5.73	682	5.73	703	5.73	Function unknown
W	15	0.13	15	0.13	15	0.12	Extracellular structure
Z	1	0.008	1	0.008	1	0.008	Cytoskeleton

^a^The total is based on the total number of protein encoding genes in the genome

**Fig. 4:**
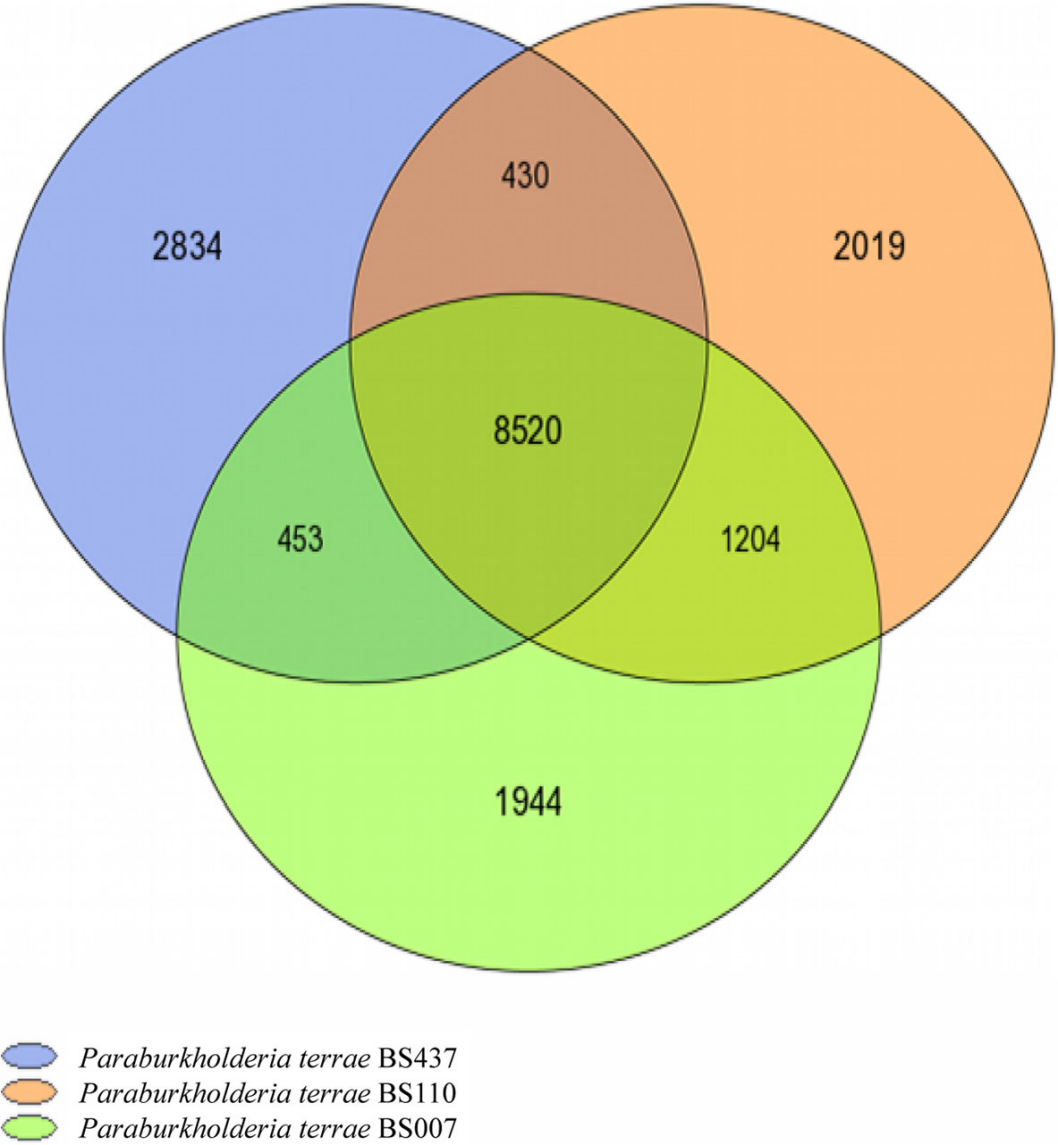
Core and pan genomes. Venn diagram analysis of 10.1601/nm.27008 strain BS007, strain BS110, and strain BS437

Comparative genomics based analyses of the pan and core genomes of strains BS007, BS110 and BS437 revealed that these - across the three strains - comprised 17,404 coding regions, whereas the core genome contained only 8520 such regions. The variable genome thus contained 8884 coding regions. The analysis further showed that the three strains contain 15.79%, 16.26% and 22.75% strain-specific coding regions, respectively (Fig. 4; Additional file 1: Table S2).

## Insights into the genome sequences

Each of the genomes of 10.1601/nm.27008 strains BS007, BS110 and BS437 was found to contain genes predicted to encode highly diverse primary and secondary metabolisms, as previously found in strain BS001 [8]. For example, numerous sets of genes were predicted to be involved in carbohydrate metabolism (Additional file 1: Table S3). Also, genes for predicted uptake systems were abundantly present across the three strains. Remarkably, the glycerol uptake and glycerol kinase genes *glpK* and *glpD* were found consistently across all three strains. These genes had 100% homology with the same genes found in strain BS001. Secondary metabolite analyses showed that the three strains contain 14, 16 and 17 gene clusters encoding these (strain BS007, BS110 and BS437, respectively; Additional file 1: Table S4). In each strain, one gene cluster was found for non-ribosomal peptide synthetase (NRPS) and a hybrid NRPS and polyketide synthase (PKS). Remarkably, the NRPS-PKS encoding systems of strains BS007 and BS110 had the same length (12,267 bp) as well as peptide monomer composition (val-mal-gly). In contrast, the strain BS437 system was shorter (length 9398 bp) and had a reduced peptide monomer composition (mal-gly). Remarkable, we found an additional NRPS gene cluster, uniquely, in the genome of strain BS110 (Additional file 1: Table S4). Next to these gene clusters, others encoding bacteriocin, terpene, ectoine, phosphonate and aryl polene production were also found in all three strains (Additional file 1: Table S4).

In addition, sets of plant-interactive genes were detected in all three genomes. In particular, those for production of indole acetic acid from tryptophan,  as well as of 1-aminocyclopropane-1-carboxylate deaminase (ACC deaminase), were found.  We also found the nodulation genes *nod* *I*, *nod* *J*, *nod* *N* and *nod* *W* across all three genomes, next to (uniquely) *nod* *V* in strain BS110 (Additional file 1: Table S5). Similar sets of genes have previously been found in strain BS001 and these were implied in a putative ‘rhizosphere phase’ of this strain [8]. Together, the data indicated the presence of genes for a convergent suite of traits with ecological relevance across the three strains.

With respect to fungal interactivity, the bacterial type-4 pilus system might be involved [14]. In 10.1601/nm.2553, type-4 pili are required for microbial motility as well as biofilm adherence [15]. In our three strains, we found complete sets of type-4 pili genes, named *pilA, pilB, pilC, pilD, pilF, pilM, pilN, pilO/pilP, pilQ, pilT* and *fimT* (Table 7). This gene constellation is, however, different from that of strain BS001, which apparently lost its *pilP* gene [14].

**Table 7:** Presumed plant- and fungal-interactive traits in *Paraburkholderia terrae* strain BS110, BS007, and BS437

Strain	Traits^a^							
	Plant-interactive	Fungal-interactive						
		T2SS	T3SS	T4SS	T6SS	T4P	Biofilm formation	Glycerol uptake and metabolism
BS007	+	+	+	+	+	+	+	+
BS110	+	+	+	+	+	+	+	+
BS437	+	+	+	+	+	+	+	+

^a^Indicates the presence of plant- and fungal-interactive traits; abbreviations in glossary. For more details see Additional file 1: Tables S6-S8

The ability of bacteria to produce exopolysaccharides is critical in biofilm formation, and the biofilm (extra-matrix) poly-β-1,6-N-acetyl-D-glucosamine (PGA) system has been shown to be an important component of 10.1601/nm.26956 biofilms [16]. PGA-encoding genes were previously found in the strain BS001 genome [8]. Other exopolysaccharide-production systems, such as those for alginate, *pel* and *psl,* have been identified in 10.1601/nm.2553 [17]. The analysis of the genomes of the three novel strains uncovered several such systems in all strains. Specifically, complete PGA systems (*pgaA, pgaB, pgaC* and *pgaD*), next to two genes of the pel (*pelB* and *pelD*) system, were found. In 10.1601/nm.2553, the *pel* (*pelA-F*) system produces a biofilm matrix, a glucose-rich polysaccharide polymer that has essential structural and protective roles [18]. The analysis also found several alginate production system genes (*algA, algB, algC, algD, algP, algU* and *kinB*) in all strains. The exception was *algE1*, which was only found in the strain BS007 genome. In contrast, we did not find any gene from the *psl* exopolysaccharide production system (Table 7).

**Fig. 5:**
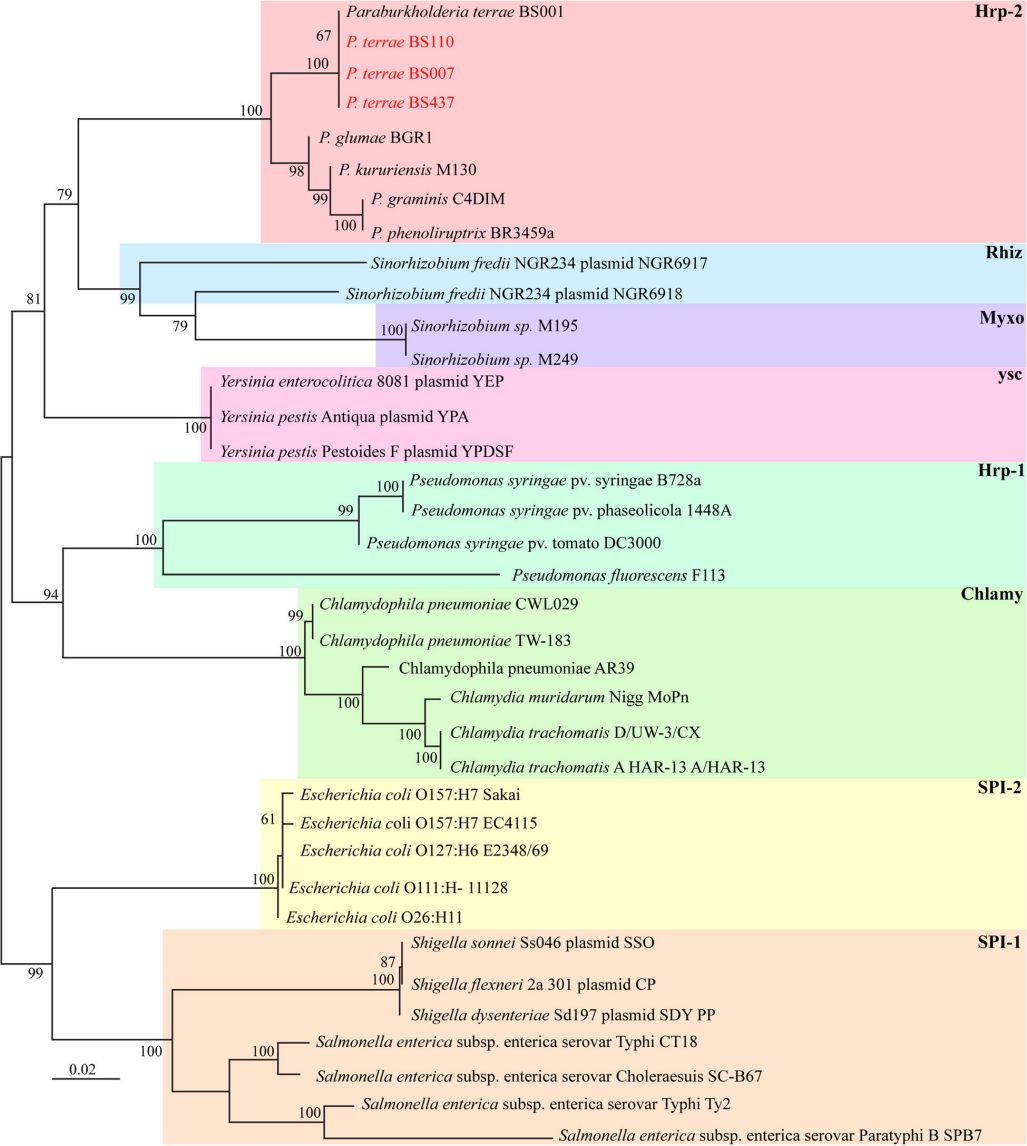
Phylogenetic tree of selected type-3 secretion systems (T3SS). The tree was generated based on alignment of eight conserved genes of the T3SS (*SctS, SctR, SctQ, SctV, SctU, SctJ, SctN,* and *SctT*). Evolutionary distance was computed with MEGA7 using a maximum-likelihood method. The bootstrap values above 50% (from 1000 replicates) are indicated at the nodes. The T3SSs of 10.1601/nm.27008 strains BS007, BS110 and BS437 T3SS belong to the Hrp-2 type, as previously reported for BS001 [8]. Different types of T3SSs were described in Abby and Rocha [19]

**Fig. 6:**
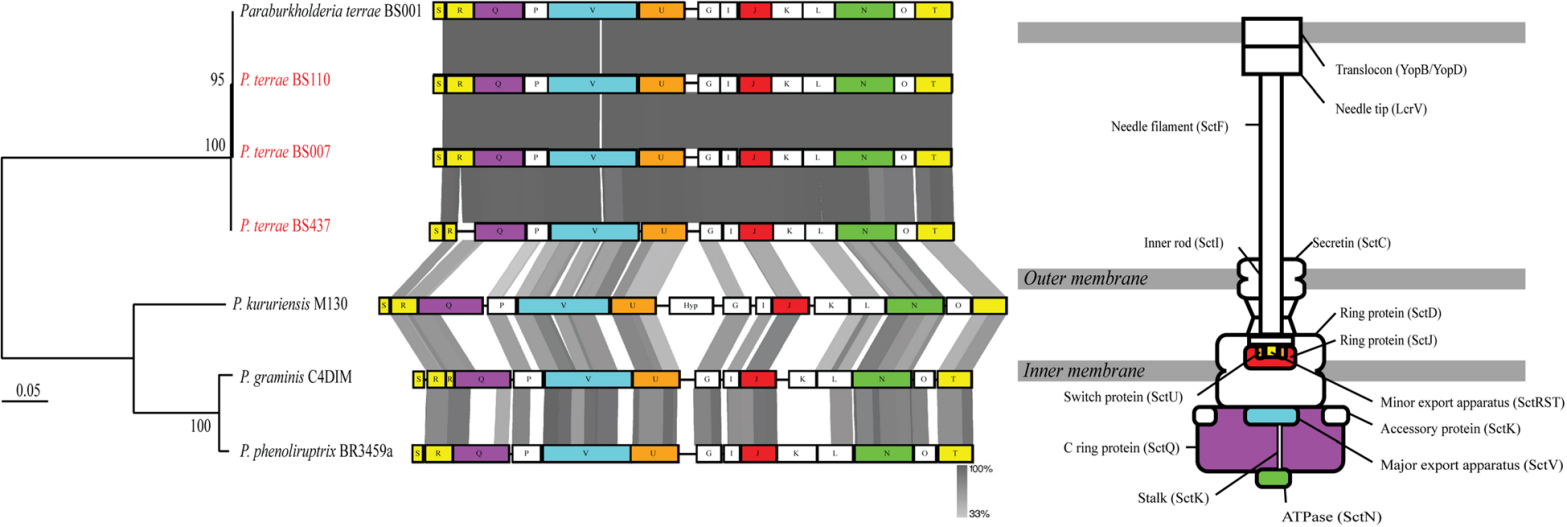
Synteny comparison of Hpr-2 type-3 secretion systems (T3SS) used in the phylogenetic tree. Evolutionary distance was computed with MEGA7 using a maximum-likelihood method. The bootstrap values above 50% (from 1000 replicates) are indicated at the nodes. The tree was generated based on alignment of eight conserved genes of the T3SS (*SctS, SctR, SctQ, SctV, SctU, SctJ, SctN,* and *SctT*) indicated by the colored boxes. The letter in the boxes indicates the last letter of the *Sct* gene. Comparison percentage was generated using BLAST+ 2.4.0 (tBLASTx with cutoff value 10^−3^) and map comparison figures were created with the Easyfig program [33]

Furthermore, complete sets of T3SS-encoding genes were found in all three genomes (Table 7). A phylogenetic tree based on eight (concatenated) conserved genes (*SctS, SctR, SctQ, SctV, SctU, SctJ, SctN* and *SctT*) of the T3SS showed that all systems belong to the Hrp-2 type of the T3SS (Figs. 5 and 6). It has been suggested that this type is required for the establishment of interaction with fungi [19, 20]. Moreover, copies (sometimes partial) of other secretion systems, i.e. the T1SS, T2SS, T4SS and T6SS, were discovered in the three genomes (Additional file 1: Table S6). These genomic evidences indicate that the three 10.1601/nm.27008 strains are highly versatile in a range of (potentially host-related) niches in soil.

**Fig. 7:**
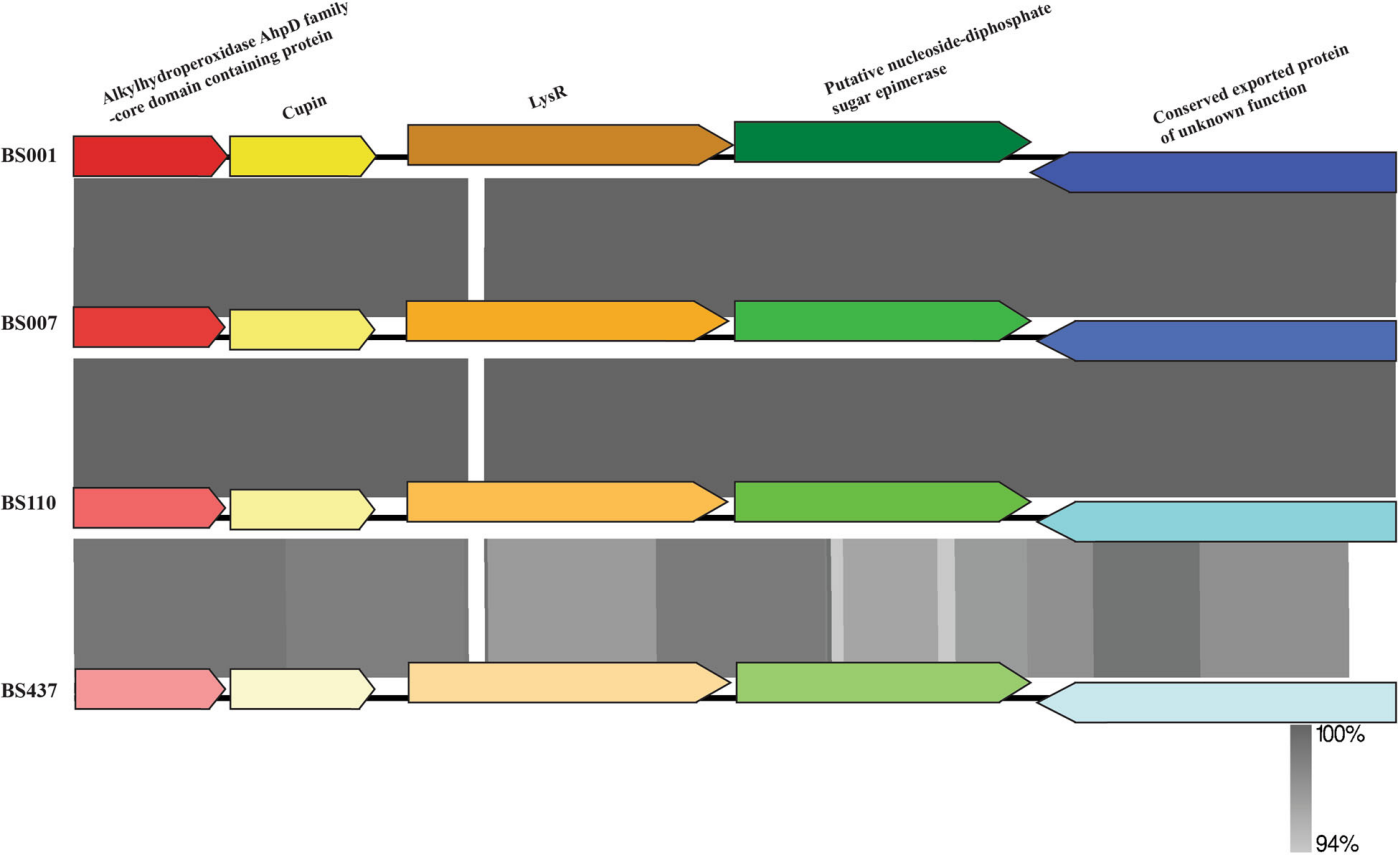
Synteny comparison five-gene cluster among strains. The corresponding genes were indicated by the color boxes. Comparison percentage was generated using BLAST+ 2.4.0 (tBLASTx with cutoff value 10^−3^) and figures were created with the Easyfig program [33]

We previously found that, upon physical contact with the soil fungus *L*. sp strain Karsten, a five-gene cluster in 10.1601/nm.27008 strain BS001 becomes highly expressed [12]. This gene cluster was hypothesized to be involved in energy generation coupled to an oxidative stress response, with four of the five genes being highly upregulated [12]. The five-gene cluster includes an alkyl hydroperoxidase *AhpD* family core domain containing protein, a cupin domain containing protein, a *LysR* family transcriptional regulator, a putative nucleoside-diphosphate sugar epimerase and a conserved exported protein of unknown function [12]. Our current genome analyses revealed that the complete gene cluster was present in all of the newly sequenced genomes (Additional file 1: Table S7). A synteny assessment of the respective clusters of the strain BS007, BS110 and BS437 genomes with that of strain BS001 showed synteny and  high levels of homology across all clusters (94%–100%) (Fig. 7).

### Presence of bacteriophage-related sequences

We finally analyzed the three genomes for the presence of prophage-like sequences, as prophages endow bacteria with traits that may advance their evolutionary fitness (following a lysogenic conversion). Thus, phenotypic plasticity of the host bacteria (i.e. with respect to virulence factors, auxiliary metabolic genes, and traits affecting biofilm formation) is fostered [21–23]. The analyses showed that the genomes of 10.1601/nm.27008 BS110, BS007 and BS437 all contain considerable amounts of prophage-like sequences (9.9%, 11.8% and 11.3%, respectively), with strain BS437 being able to produce phage progeny [34].

We then analyzed the three genomes for the presence of CRISPR-Cas spacer sequences. CRISPR-Cas systems provide so-called adaptive immunity to bacteria, serving as a heritable record of past infections with phages or other extraneous elements [24]. Using the (web-based) CRISPRFinder program [25], we found CRISPR sequences to be present in all three strains; respectively 21, 22 and 15 such sequences were found in strains BS007, BS110 and BS437. This finding indicated the host strains had been exposed to numerous extrachromosomal element (e.g. phage) infestations.

## Conclusions

The here reported genome analyses of the fungal-interactive 10.1601/nm.27008 strains BS110, BS007 and BS437 revealed that all genomes were large in size, encompassing a suite of metabolic, nutrient capture and ‘interactivity’ genes. The repertoire of genetic systems found probably encompasses traits that allow adaptation to niches in the soil as influenced by organisms such as fungi, as well as plants. Moreover, potential defense systems were also found. Thus, all genomes harbored highly diverse primary and secondary metabolite systems. Furthermore, they also contained sets of genes for type-4 pili, biofilm formation (PGA, alginate and *pel*), secretion systems (T1SS, T2SS, T3SS, T4SS and T6SS) and glycerol uptake systems; such systems potentially enable them to reap the ecological benefits conferred by fungal hyphae in soil. A five-gene cluster, that had been found to be highly upregulated upon physical contact with *Lyophyllum* sp. strain Karsten in strain BS001, was consistently found in all three strains. This allowed the hypothesis that this gene cluster may confer a fitness advantage to the organisms in the early stages of contact with fungal mycelium in soil. Finally, our analyses also highlight the presence of a considerable amount of prophage-like sequences, complete or incomplete, in the 10.1601/nm.27008 genomes. The significance of these prophage sequences for the host cells and their effects on the ecological functioning and adaptability of the hosts is still under investigation.

## Additional file

Additional file 1:
**Table S1** Sequencing statistics analysis of *Paraburkholderia terrae* BS007, BS110, and BS437. **Table S2** Pan/core genome. **Table S3** Metabolic profiles of compared bacterial strains (Based on a score from 0 to 1). **Table S4.1** Secondary metabolites, BS007. **Table S4.2** Secondary metabolites, BS110. **Table S4.3** Secondary metabolites, BS437. **Table S5.1** Nodulation genes. **Table S5.2** Type 4 pili and biofilm formation systems. **Table S5.3** Indole acetic acid (IAA) biosynthesis. **Table S5.4** 1-aminocyclopropane-1-carboxylate deaminase (ACC deaminase). **Table S6.1** Type-1 secretion systems. **Table S6.2** Type-2 secretion systems. **Table S6.3** Type-3 secretion systems. **Table S6.4** Type-4 secretion systems. **Table S6.5** Type-6 secretion systems. **Table S7** Homology of putative energy-generating gene clusters to *P. terrae* BS001. (XLSX 164 kb)

## Abbreviations

CRISPR: Clustered regularly interspaced short palindromic repeats
T1SS: type 1 secretion system
T2SS: type 2 secretion system
T3SS: type 3 secretion system
T4P: type 4 pili
T4SS: type 4 secretion system
T6SS: type 6 secretion system
